# Supplementary material for: Molecular landscape and functional characterization of centrosome amplification in ovarian cancer
Source: Nat Commun. 2023 Oct 16;14:6505. doi: 10.1038/s41467-023-41840-3 (PMC10579337; doi:10.1038/s41467-023-41840-3)
Supplement: Supplementary file 1 — Supplementary Information [file 41467_2023_41840_MOESM1_ESM.pdf]

Supplementary Information

Molecular landscape and functional characterization of centrosome amplification in ovarian cancer

Carolin M. Sauer[1,2,$], James A. Hall[1,2], Dominique-Laurent Couturier[1,3], Thomas Bradley[1,2], Anna M. Piskorz[1,2], Jacob Griffiths[1,2], Ashley Sawle[1,2], Matthew D. Eldridge[1,2], Philip Smith[1,2], Karen Hosking[2], Marika A.V. Reinius[1,2,4], Lena Morrill Gavarró[1,2], Anne-Marie Mes-Masson[5], Darren Ennis[6], David Millan[7], Aoisha Hoyle[8], Iain A. McNeish[6], Mercedes Jimenez-Linan[4], Filipe Correia Martins[1,4], Julia Tischer[1,2], Maria Vias[1,2], James D. Brenton[1,2,4,$]

1. Cancer Research UK Cambridge Institute, University of Cambridge, Li Ka Shing Centre, Robinson Way, Cambridge CB2 0RE, UK.
2. Cancer Research UK Major Centre–Cambridge, University of Cambridge, Cambridge CB2 0RE, UK.
3. Medical Research Council Biostatistics Unit, University of Cambridge, Cambridge CB2 0SR, UK.
4. Cambridge University Hospital NHS Foundation Trust and National Institute for Health Research Cambridge Biomedical Research Centre, Addenbrooke's Hospital, Cambridge, UK.
5. Department of Medicine, Université de Montréal and Centre de recherche du Centre hospitalier de l'Université de Montréal (CRCHUM), Montreal, QC, Canada.
6. Department of Surgery and Cancer, Ovarian Cancer Action Research Centre, Imperial College London, London, UK.
7. Department of Pathology, Queen Elizabeth University Hospital, Glasgow, UK.
8. Deparment of Pathology, University Hospital Monklands. NHS Lanarkshire, UK.

[$]Correspondence: carolin.sauer@cruk.cam.ac.uk; james.brenton@cruk.cam.ac.uk

Table of Contents

Supplementary Figures **2**

Supplementary Figure 1 – Spatial heatmaps of centrosome amplification 2
Supplementary Figure 2 – CA20 gene expression signature is not associated with survival in high CIN cancers. 4
Supplementary Figure 3 – Differential gene expression analysis in ovarian cancer vs. normal cell lines. 5
Supplementary Figure 4 – Centrosome amplification is not associated with distinct genomic features of copy number aberrations in HGSOC tissues. 6
Supplementary Figure 5 – Genomic landscape of ovarian cancer cell lines. 7
Supplementary Figure 6 – Correlation of CA and MN with genomic features in ovarian cancer cell lines. 9

Supplementary Tables **10**

Supplementary Table 1 – Overview of patients and tissue samples included in centrosome staining study. 10
Supplementary Table 2 – Cell line information and culture growth conditions. 11
Supplementary Table 3 – Primary antibodies for immunofluorescent staining of cell lines. 14

Supplementary Methods – Method Development **15**

Optimisation of immunofluorescent centrosome staining in clinical FFPE tissues 15
High-throughput microscopy image acquisition and analysis 17

Supplementary References **20**

Supplementary Figures

Supplementary Figure 1 – Spatial heatmaps of centrosome amplification

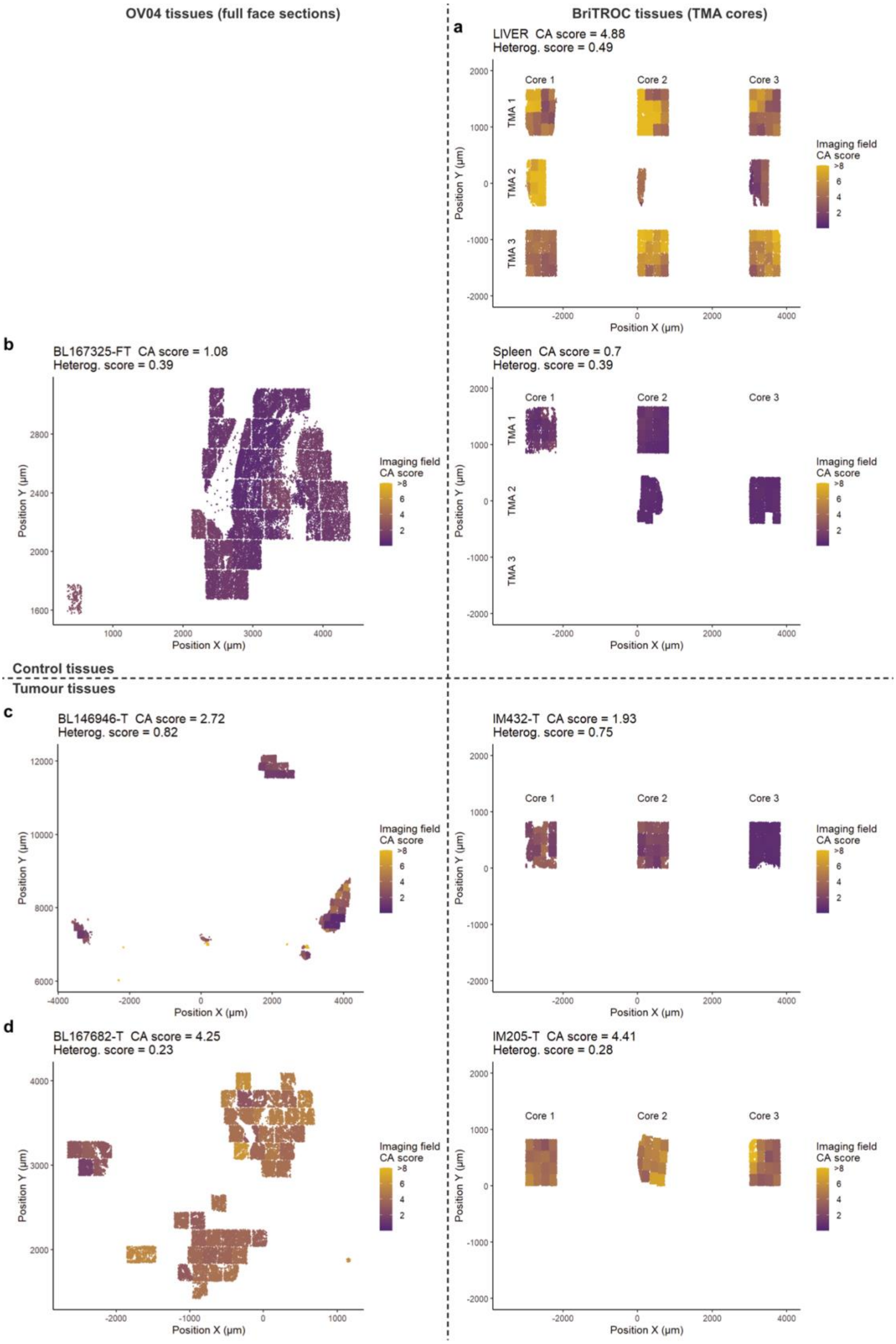

Examples from the OV04 cohort are shown on the left panel, examples from the BriTROC cohort are shown on the right panel. (**a**) Positive control tissue (Liver). (**b**) Negative control (normal) tissues – Fallopian tube on the left,

spleen on the right. (**c**) HGSOC tissues with low to mid-range CA and high tissue heterogeneity. (**d**) HGSOC tissues with high CA and low tissue heterogeneity. CA scores are indicated by a colour gradient (purple = low, yellow = high) for each imaging field. Points represent individual nuclei detected during image analyses and were plotted in relation to their physical position (μm) on the microscope slide (x and y axis). *Note that CMYK printing may obscure differences on the CA score colour scale.*

Supplementary Figure 2 – CA20 gene expression signature is not associated with survival in high CIN cancers.

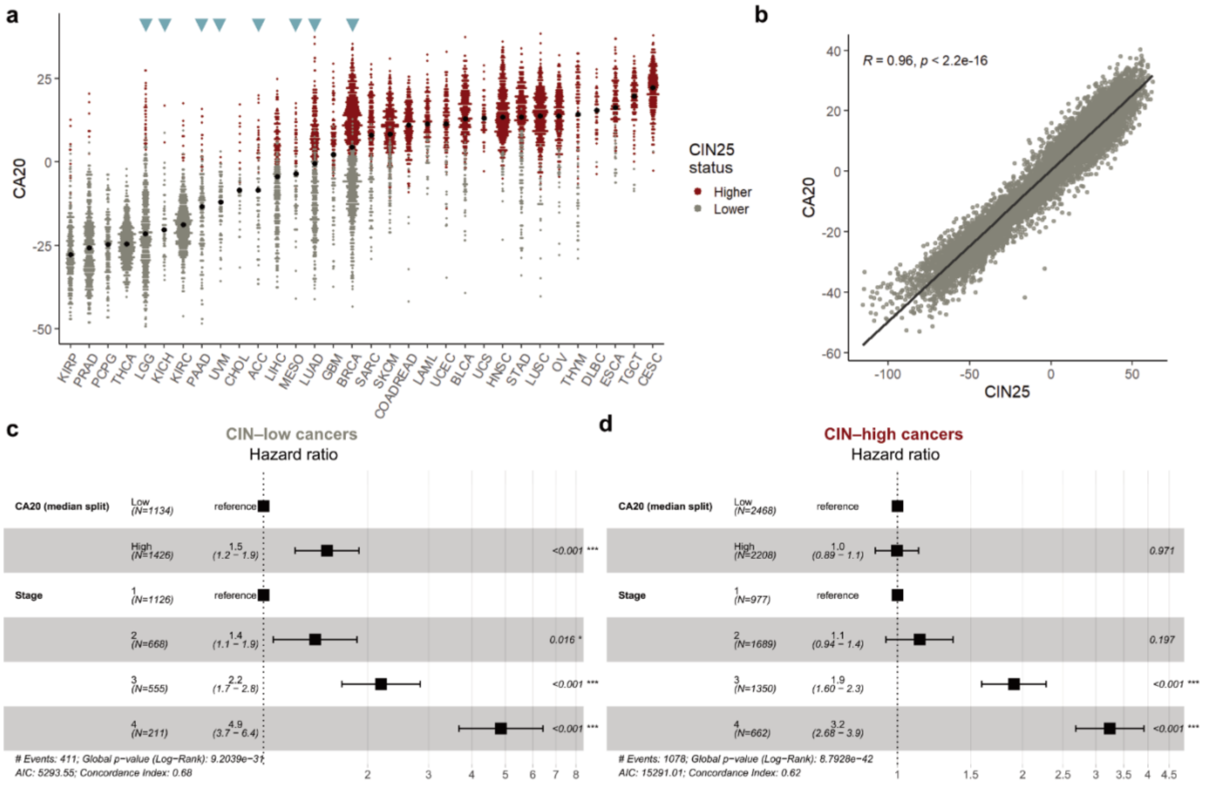

(**a**) CA20 gene expression scores across TCGA pan-cancers (sorted by median CA20 scores indicated by black dots). Each point indicates an individual tumour sample colour coded by the CIN25 gene expression status (median split; red = high, grey = low). Cancer subtypes highlighted by blue arrowheads are cancers in which CA has previously been implicated in poor survival outcome[1] (**b**) Spearman's rank correlation (two-sided) of CA20 and CIN25 signature expression (n=9602 TCGA tumour samples). (**c**) and (**d**) Forest plots of multivariable Cox proportional hazard modelling on overall survival for low-CIN (n=2560) and high-CIN (n=4676) cancers, respectively. Squares display the hazard ratio (HR) and whiskers the 95% confidence intervals of the HR. p-values shown are derived from likelihood ratio tests.

Supplementary Figure 3 – Differential gene expression analysis in ovarian cancer vs. normal cell lines.

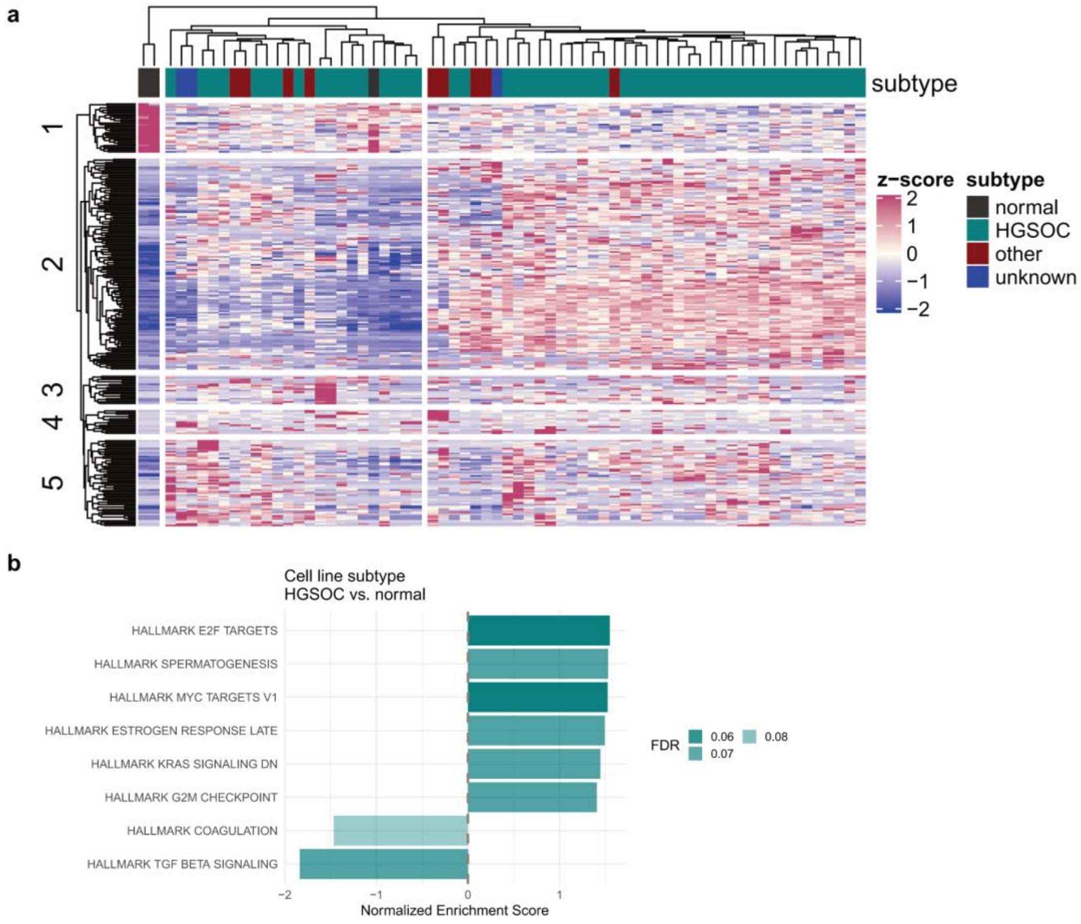

(**a**) Gene expression heatmap across different histological subtypes showing the top 250 differentially expressed genes in HGSOC vs. normal cell lines (FT246, FT194 and IOSE4). Genes and cell lines were grouped using hierarchical clustering. Subtypes are indicated by different colours. (**b**) Gene set enrichment analysis (GSEA) of differentially expressed genes in HGSOC vs. normal cell lines, showing all pathways for which $p < 0.05$ and $FDR < 0.1$. Gene set enrichment analysis and testing was performed using the fgsea R package.

Supplementary Figure 4 – Centrosome amplification is not associated with distinct genomic features of copy number aberrations in HGSOC tissues.

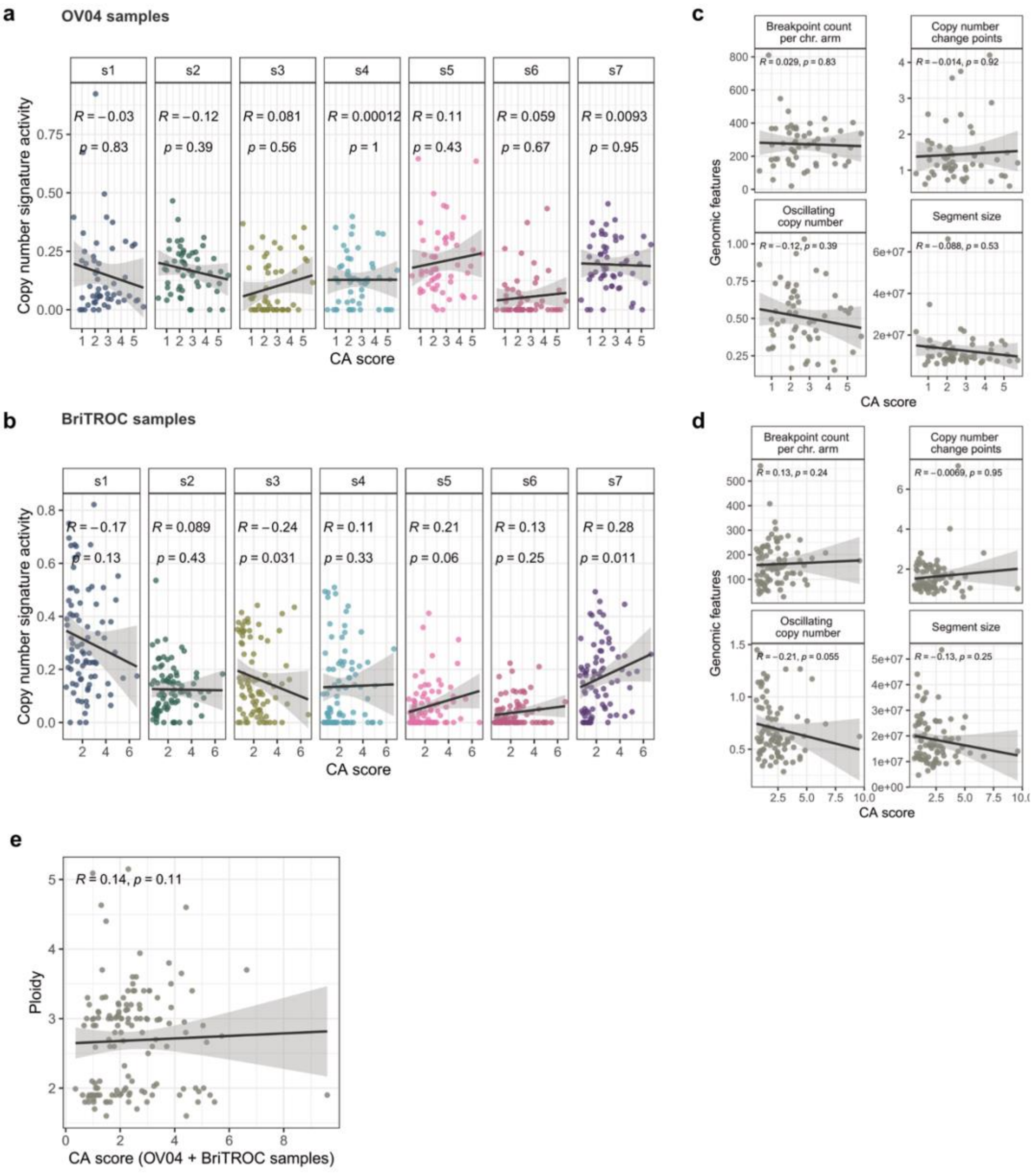

Correlation of copy number signature exposures and CA for (**a**) OV04 samples, and (**b**) BriTROC samples. (**c**) and (**d**) show correlations between CA scores and genomic features for OV04 and BriTROC samples, respectively. (**e**) Correlation of CA scores (from both BriTROC and OV04 samples) and estimated ploidies (a-e, n=54 OV04 and n=84 BriTROC samples). Coefficients were estimated using Spearman's rank correlations (two-sided). Error bands show 95% confidence intervals. Note that in the BriTROC cohort, there was a negative correlation of CA scores with signature 3, and a positive correlation with signature 7. However, no significant associations were observed following p-value adjustment for multiple comparisons.

Supplementary Figure 5 – Genomic landscape of ovarian cancer cell lines.

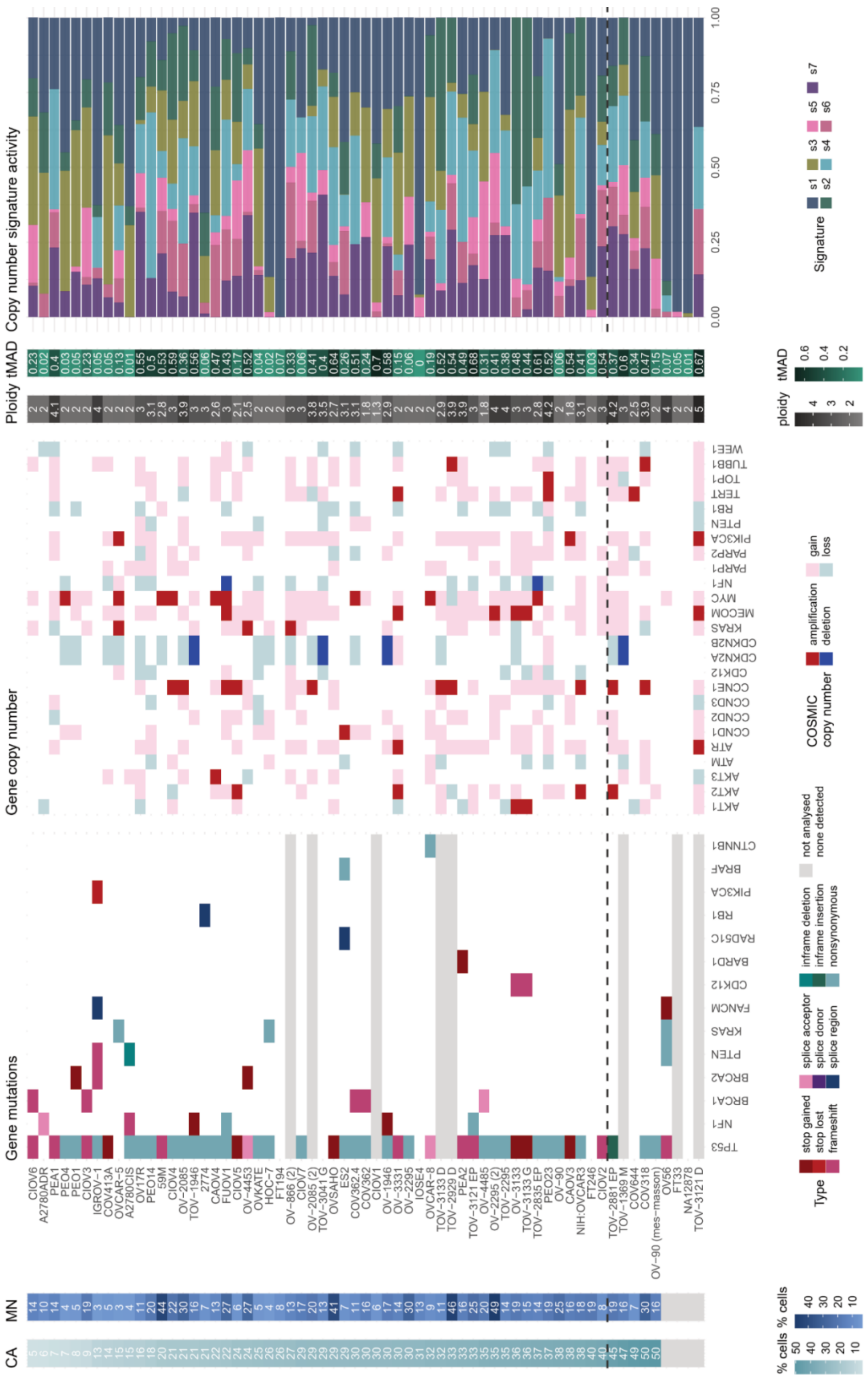

Cell lines are displayed and ordered by their mean centrosome amplification (CA) frequencies (panel 1). Micronuclei (MN) frequencies are plotted along CA frequencies (panel 2). Panel 3 shows an oncoprint plot of gene mutations called from TAm-Seq data. Synonymous and non-pathogenic nonsynonymous mutations or

nonsynonymous mutation of unknown clinical significance were excluded. Panel 4 shows gene copy number states (amplification, deletion, gain or loss) for HGSOC genes of interest. Amplifications and deletions were called using the definitions outlined by the Catalogue of Somatic Mutations in Cancer (COSMIC). Amplifications: ploidy ≤2.7 and CN ≥5; or ploidy >2.7 and CN ≥9. Deletion: ploidy ≤2.7 and CN = 0; or ploidy >2.7 and CN < (ploidy − 2.7). CN gain and losses are defined as a CN change of +1 or −1 from a sample's ploidy, respectively. Ploidies are indicated in panel 5. Trimmed median absolute deviation from CN neutrality (tMAD) scores are shown in panel 6. Panel 7 shows the copy number signature activities derived from ACN-fitted sWGS data for each cell line. Across all cell lines, the most frequently mutated gene was *TP53* (82%), followed by *NF1* (11%), *BRCA1* (9%) and *BRCA2* (5%). ACN fitting was performed on the sWGS data, and copy number states (amplification, gain, loss and deletion) were called for HGSOC genes of interest depicted in panel 4. The median ploidy across all analysed cell lines was 2.9. The most frequently amplified gene was *CCNE1* (n= 10), followed by *MYC* (n = 9) and *MECOM* (n = 7), while the most frequently gained genes were *MECOM* (n = 30), *PIK3CA* (n = 28) and *MYC* (n = 27). By contrast, copy number deletions were only observed for *CDKN2A*/*B* in four cell lines, and *NF1* in two cell lines. The most frequently lost genes were *RB1* (n = 16), *CDKN2A*/*B* (n = 15) and *NF1* (n = 12). Together, these results suggest that the analysed cell lines are broadly representative of the overall HGSOC case population[2,3].

Supplementary Figure 6 – Correlation of CA and MN with genomic features in ovarian cancer cell lines.

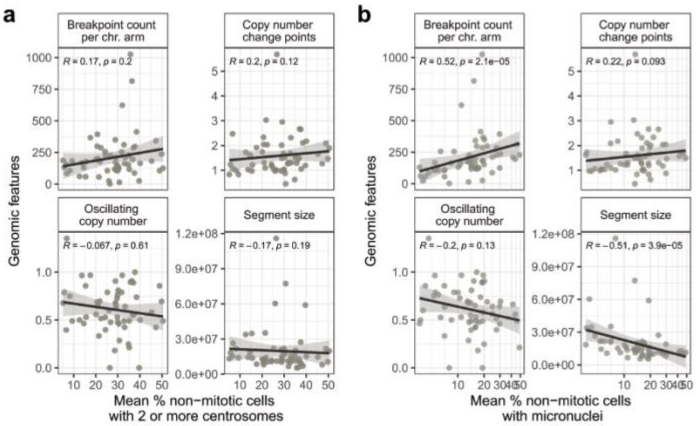

Correlations of genomic features with (**a**) centrosome amplification (CA) and (**b**) micronuclei (MN) frequencies. Coefficients were estimated using Spearman's rank correlations (two-sided). Error bands show 95% confidence intervals. N=59 cell lines (for which matching sWGS and imaging data was available).

Supplementary Tables

Supplementary Table 1 – Overview of patients and tissue samples included in centrosome staining study.

		OV04 cohort	BriTROC cohort
Total number of patients		85	141
Total number of tumour tissues		93	194
Age at diagnosis	Median	65	65
	Range	44–83	24–85
Histotype	HGSOC	85 (100%)	136 (96%)
	Endometrioid		4 (3%)
	Unknown		1 (1%)
Figo substage	I	4 (5%)	10 (7%)
	II	3 (4%)	15 (11%)
	III	47 (55%)	90 (64%)
	IV	27 (32%)	26 (18%)
	Unknown	4 (5%)	
Surgery type	Immediate primary surgery (IPS)	25 (29%)	92 (65%)
	Delayed primary surgery (DPS)	57 (67%)	43 (30%)
	Unknown	3 (4%)	6 (4%)
Survival (years)	Median	2.4	4.9
	Range	0.26–7.7	0.76–16.6
Sample type	Full face section	93 (100%)	
	Cores in tissue microarray (TMA)		194 (100%)

Overview table of OV04 and BriTROC patients and associated characteristics of samples included in tissue staining experiments for centrosome detection. TMA = tissue microarray; IPS = Immediate primary surgery; DPS = delayed primary surgery.

Supplementary Table 2 – Cell line information and culture growth conditions.

CellLine	RRID	Media	Supplements	Oxygen	Provider	Type
**2774**		DMEM/F-12	10% FBS	21%	Jozien Helleman, Netherlands	OV endometrioid carcinoma
**59M**	CVCL_2291	DMEM/F-12	10% FBS	21%	Jozien Helleman, Netherlands	HGSOC
**A2780**	CVCL_0134	RPMI 1640	10% FBS + 2mM Sodium Pyruvate	21%	Jozien Helleman, Netherlands	unknown
**A2780ADR**	CVCL_1941	RPMI 1640	10% FBS + 2mM Sodium Pyruvate	21%	Jozien Helleman, Netherlands	unknown
**A2780CIS**	CVCL_1942	RPMI 1640	10% FBS + 2mM Sodium Pyruvate	21%	Jozien Helleman, Netherlands	unknown
**CAOV3**	CVCL_0201	DMEM/F-12	10% FBS	21%	unknown	HGSOC
**CAOV4**	CVCL_0202	L-15	10% FBS	21%	Jozien Helleman, Netherlands	HGSOC
**CIOV1**		DMEM/F-12	10% FBS	21%	developed in-house; Maria Vias	HGSOC
**CIOV2**		DMEM/F-12	10% FBS	21%	developed in-house; Maria Vias	HGSOC
**CIOV3**		DMEM/F-12	10% FBS	21%	developed in-house; Maria Vias	HGSOC
**CIOV4**		DMEM/F-12	10% FBS	21%	developed in-house; Maria Vias	HGSOC
**CIOV5**		DMEM/F-12	10% FBS	21%	developed in-house; Maria Vias	HGSOC
**CIOV6**		DMEM/F-12	10% FBS	21%	developed in-house; Maria Vias	HGSOC
**CIOV7**		DMEM/F-12	10% FBS	21%	developed in-house; Maria Vias	HGSOC
**COV318**	CVCL_2419	DMEM/F-12	10% FBS	21%	Jozien Helleman, Netherlands	HGSOC
**COV362**	CVCL_2420	DMEM/F-12	10% FBS	21%	Jozien Helleman, Netherlands	OV endometrioid carcinoma
**COV362.4**	CVCL_2421	DMEM/F-12	10% FBS	21%	Jozien Helleman, Netherlands	OV endometrioid carcinoma
**COV413A**	CVCL_2422	DMEM/F-12	10% FBS	21%	Jozien Helleman, Netherlands	OV epithelial-serous carcinoma
**COV644**	CVCL_2425	DMEM/F-12	10% FBS	21%	Jozien Helleman, Netherlands	OV epithelial-mucinous carcinoma
**ES2**	CVCL_3509	McCoy's 5A	10% FBS	21%	unknown	HGSOC
**FT194**	CVCL_UH58	DMEM/F-12	10% FBS	21%	Ronnie Drapkin	normal fallopian tube
**FT246**	CVCL_UH61	DMEM/F-12	10% FBS	21%	Ronnie Drapkin	normal fallopian tube
**FT33**	CVCL_RK66	DMEM/F-12	10% FBS	21%	Ronnie Drapkin	normal fallopian tube
**FUOV1**	CVCL_2047	DMEM/F-12	10% FBS	21%	DSMZ, ACC444, Lot7, 7.03.2018	HGSOC
**HeLa**	CVCL_0030	DMEM/F-12	10% FBS	21%	ATCC	endocervical adenocarcinoma
**HOC-7**	CVCL_5455	RPMI 1640	10% FBS + 2mM Sodium Pyruvate	21%	Jozien Helleman, Netherlands	LGSOC
**IGROV1**	CVCL_1304	RPMI 1640	10% FBS + 2mM Sodium Pyruvate	21%	Jozien Helleman, Netherlands	OV endometrioid carcinoma
**IOSE4**	CVCL_0T70	DMEM/F-12	10% FBS	21%	unknown	normal OV surface epithelium
**JHOS-2**	CVCL_4647	DMEM/F-12	10% FBS	21%	Wendy Fantl, Stanford	HGSOC
**Kuramochi**	CVCL_1345	RPMI 1640	10% FBS + 2mM Sodium Pyruvate	21%	Wendy Fantl, Stanford	HGSOC
**NA12878**	CVCL_7526	RPMI 1640	10% FBS + 2mM Sodium Pyruvate	21%	Caldas lab	lymphoblastoid
**NIH:OVCAR3**	CVCL_0465	RPMI 1640	10% FBS + 2mM Sodium Pyruvate	21%	ATCC	HGSOC
**OAW28**	CVCL_1614	DMEM/F-12	10% FBS	21%	Jozien Helleman, Netherlands	HGSOC
**OAW42**	CVCL_1615	DMEM/F-12	10% FBS	21%	Jozien Helleman, Netherlands	OV cystadenocarcinoma

Supplementary Table 2 continued.

CellLine	RRID	Media	Supplements	Oxygen	Provider	Type
**OV-1369 (2)**	CVCL_9T12	OSE	10% FBS	5%	Mes Masson, Canada	HGSOC
**OV17R**	CVCL_2672	DMEM/F-12	10% FBS	21%	Jozien Helleman, Netherlands	OV adenocarcinoma
**OV-1946**	CVCL_4375	OSE	10% FBS	5%	Mes Masson, Canada	HGSOC
**OV-2085**	CVCL_A1SI	OSE	10% FBS	5%	Mes Masson, Canada	HGSOC
**OV-2085 (2)**		OSE	10% FBS	5%	Mes Masson, Canada	HGSOC
**OV-2295**	CVCL_9T13	OSE	10% FBS	5%	Mes Masson, Canada	HGSOC
**OV-2295 (2)**	CVCL_9T14	OSE	10% FBS	5%	Mes Masson, Canada	HGSOC
**OV-2978**	CVCL_A1SM	OSE	10% FBS	5%	Mes Masson, Canada	HGSOC
**OV-3133**	CVCL_9T15	OSE	10% FBS	5%	Mes Masson, Canada	HGSOC
**OV-3133(2)**	CVCL_9T16	OSE	10% FBS	5%	Mes Masson, Canada	HGSOC
**OV-3331**	CVCL_A1SQ	OSE	10% FBS	5%	Mes Masson, Canada	HGSOC
**OV-4453**	CVCL_9T20	OSE	10% FBS	5%	Mes Masson, Canada	HGSOC
**OV-4485**	CVCL_9T21	OSE	10% FBS	5%	Mes Masson, Canada	HGSOC
**OV56**	CVCL_2673	DMEM/F-12	10% FBS	21%	Jozien Helleman, Netherlands	HGSOC
**OV-866 (2)**	CVCL_9T22	OSE	10% FBS	5%	Mes Masson, Canada	HGSOC
**OV-90**	CVCL_3768	OSE	10% FBS	21%	unknown	HGSOC
**OV-90 (mes-masson)**	CVCL_3768	OSE	10% FBS	5%	Mes Masson, Canada	HGSOC
**OVCAR-5**	CVCL_1628	DMEM/F-12	10% FBS	21%	canadian ovarian tissue bank	unknown
**OVCAR-8**	CVCL_1629	RPMI 1640	10% FBS + 2mM Sodium Pyruvate	21%	canadian ovarian tissue bank	HGSOC
**OVKATE**	CVCL_3110	RPMI 1640	10% FBS + 2mM Sodium Pyruvate	21%	unknown	HGSOC
**OVSAHO**	CVCL_3114	RPMI 1640	10% FBS + 2mM Sodium Pyruvate	21%	Simon Langdon	HGSOC
**PEA1**	CVCL_2682	RPMI 1640	10% FBS + 2mM Sodium Pyruvate	21%	Simon Langdon	HGSOC
**PEA2**	CVCL_2683	RPMI 1640	10% FBS + 2mM Sodium Pyruvate	21%	Simon Langdon	HGSOC
**PEO1**	CVCL_2686	RPMI 1640	10% FBS + 2mM Sodium Pyruvate	21%	Simon Langdon	HGSOC
**PEO14**	CVCL_2687	RPMI 1640	10% FBS + 2mM Sodium Pyruvate	21%	Simon Langdon	HGSOC
**PEO16**	CVCL_2688	RPMI 1640	10% FBS + 2mM Sodium Pyruvate	21%	Simon Langdon	HGSOC
**PEO23**	CVCL_2689	RPMI 1640	10% FBS + 2mM Sodium Pyruvate	21%	Simon Langdon	HGSOC
**PEO4**	CVCL_2690	RPMI 1640	10% FBS + 2mM Sodium Pyruvate	21%	Simon Langdon	HGSOC
**PEO6**	CVCL_2691	RPMI 1640	10% FBS + 2mM Sodium Pyruvate	21%	Simon Langdon	HGSOC
**SKOV6**	CVCL_A457	RPMI 1640	10% FBS + 2mM Sodium Pyruvate	21%	Jozien Helleman, Netherlands	cervical squamous cell carcinoma
**SNU-119**	CVCL_5014	RPMI 1640	10% FBS + 2mM Sodium Pyruvate	21%	Wendy Fantl, Stanford	HGSOC
**TOV-1369 M**		OSE	10% FBS	5%	Mes Masson, Canada	HGSOC
**TOV-1946**	CVCL_4062	OSE	10% FBS	5%	Mes Masson, Canada	HGSOC
**TOV-2223 G**	CVCL_4063	OSE	10% FBS	5%	Mes Masson, Canada	HGSOC

Supplementary Table 2 continued.

CellLine	RRID	Media	Supplements	Oxygen	Provider	Type
**TOV-2295**	CVCL_9T18	OSE	10% FBS	5%	Mes Masson, Canada	HGSOC
**TOV-2835 EP**	CVCL_A1SJ	OSE	10% FBS	5%	Mes Masson, Canada	HGSOC
**TOV-2881 EP**	CVCL_A1SK	OSE	10% FBS	5%	Mes Masson, Canada	HGSOC
**TOV-2929 D**	CVCL_A1SL	OSE	10% FBS	5%	Mes Masson, Canada	HGSOC
**TOV-2978 G**	CVCL_9U73	OSE	10% FBS	5%	Mes Masson, Canada	HGSOC
**TOV-3041 G**	CVCL_9T24	OSE	10% FBS	5%	Mes Masson, Canada	HGSOC
**TOV-3121 D**		OSE	10% FBS	5%	Mes Masson, Canada	HGSOC
**TOV-3121 EP**	CVCL_A1SN	OSE	10% FBS	5%	Mes Masson, Canada	HGSOC
**TOV-3133 D**	CVCL_9T19	OSE	10% FBS	5%	Mes Masson, Canada	HGSOC
**TOV-3133 G**	CVCL_4064	OSE	10% FBS	5%	Mes Masson, Canada	HGSOC
**TOV-3291 G**	CVCL_9T25	OSE	10% FBS	5%	Mes Masson, Canada	HGSOC
**TYK-nu**	CVCL_1776	DMEM	10% FBS	21%	Wendy Fantl, Stanford	HGSOC
**TYK-nu.CP-r**	CVCL_3221	DMEM	10% FBS	21%	Wendy Fantl, Stanford	HGSOC

Supplementary Table 3 – Primary antibodies for immunofluorescent staining of cell lines.

Antibody	Species	Conjugated	Stain1	Stain2	Stain3	Stain4	Channel	Order number
Pericentrin	mouse	no	1 µg/ml		1 µg/ml	1 µg/ml	647	ab28144
Pericentrin	rabbit	no		1 µg/ml			555	ab4448
Cep164	rabbit	no			1 µg/ml		555	2227-1-AP
Centrin 3	mouse	no		1.1 µg/ml			647	H00001070-M01
pHH3	rabbit	no	1 µg/ml				555	06-570
Ck7 - 488	rabbit	yes	1 µg/ml		1 µg/ml		488	ab208273
Pan-Ck - 488	mouse	yes		1 µg/ml			488	53-9003-82
yH2AX - 488	mouse	yes				5 µg/ml	488	05-636-AF488
Hoechst			1 µg/ml	1 µg/ml	1 µg/ml	1 µg/ml	405	33342

Primary antibodies and associated species, order numbers and concentrations used for immunofluorescent staining of cell lines.

Supplementary Methods – Method Development

Optimisation of immunofluorescent centrosome staining in clinical FFPE tissues

FFPE tissues are the most common diagnostic samples available in the clinic. Profiling of centrosome abnormalities in clinical FFPE tissues, however, has previously been hindered by the limited thickness of FFPE sections which prevents imaging of whole cells[1,4], and the time-intensive and cumbersome image analysis. The present section describes the development of a reliable high-throughput immunofluorescent microscopy-based assay to unambiguously identify and quantify centrosomes in FFPE samples. This included the optimisation of primary antibody concentrations, detergent concentrations in blocking and antibody dilution buffers, thickness of tissue sections, and antigen retrieval methods. An overview of the immunofluorescent staining workflow for FFPE tissues with optimised conditions (highlighted in blue) for each step is shown in **Supplementary Fig. 7.**

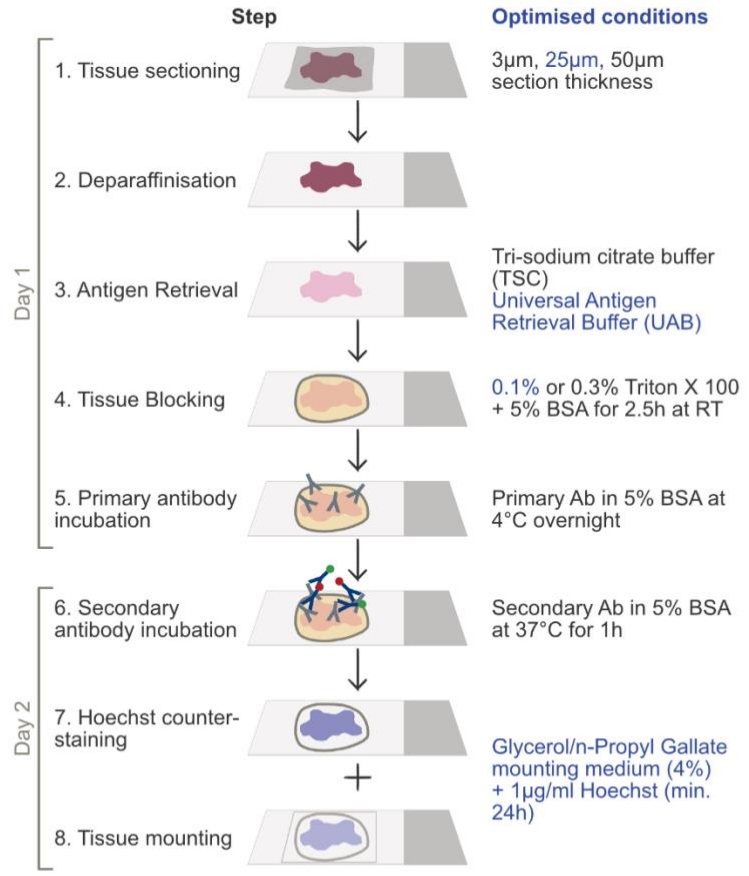

Supplementary Figure 7: Overview of the immunofluorescent staining workflow for FFPE tissue samples from tissue sectioning through to tissue mounting. Steps not shown here are the crucial washing steps before/after antibody (Ab) incubation and Hoechst staining/tissue mounting. Optimised conditions are highlighted in blue.

1. <u>Optimisation of tissue sectioning thickness</u>

To investigate the effect of tissue section thickness on centrosome detectability, tissue sections of 3µm, 25µm and 50µm thickness were stained using different antigen retrieval methods (see below). As expected, 3µm sections failed to capture intact cells resulting in centrosomes only being detected in a small fraction of cells. By contrast, sections of 50µm thickness failed to provide homogeneous staining results, owing to likely tissue penetration limitations. Using 25µm sections obviated both of these

limitations and provided homogeneous staining of mostly intact cells, facilitating the detection of centrosomes in the majority of cells.

2. <u>Optimisation of antigen retrieval methods</u>

Antigen retrieval was performed via heat-induced epitope retrieval (HIER). Two different antigen retrieval buffers were tested: Tri-Sodium Citrate (TSC) and the commercially available Universal Antigen Retrieval Buffer (UAB; ab208572). Both buffers were brought to boil using a conventional microwave. Once the boiling temperature was reached, tissue slides were immersed in either TSC or UAB buffers, boiled for 10 mins at medium-high power and subsequently incubated at room temperature for 25-30 mins. UAB-based antigen retrieval was superior to TSC-based antigen retrieval, and allowed centrosome staining and detection with both, the anti-CDK5RAP2 and anti-Pericentrin antibodies (**Supplementary Fig. 8**). Importantly, CDK5RAP2 and Pericentrin signal strongly colocalised, while no signal was observed in IgG control stains.

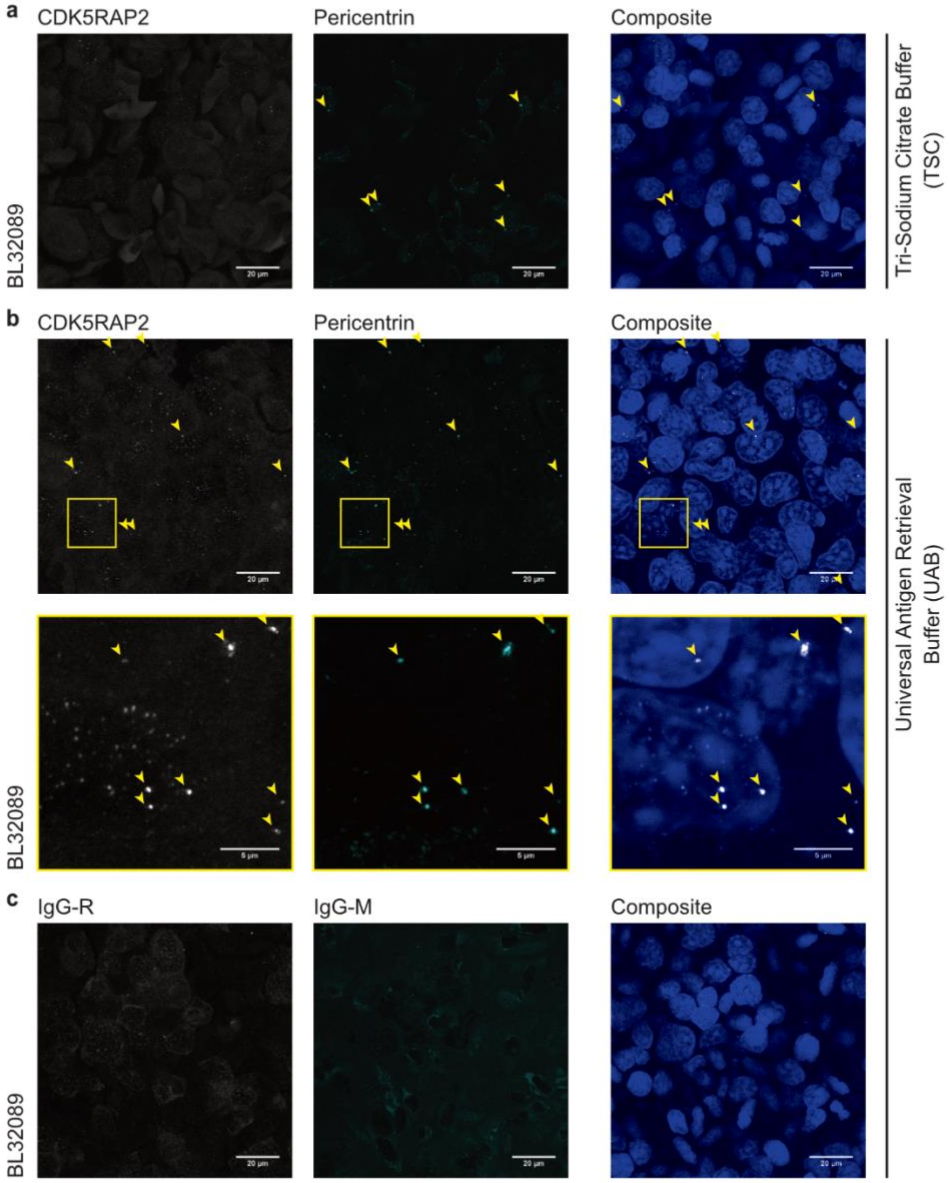

Supplementary Figure 8: The universal antigen retrieval buffer is efficient for centrosome co-staining in thicker FFPE tissue sections. Tissue sections of 25 µm thickness were co-immunolabelled with anti-CDK5RAP2 and anti-Pericentrin antibodies, and counterstained with Hoechst (blue). Confocal images (max. projections) of centrosome staining using the (**a**) Tri-Sodium Citrate (TSC) or (**b**) Universal antigen retrieval buffer (UAB). (**c**) IgG isotype control (R - rabbit; M - mouse) staining. Scale bar = 20 µm. Zoomed-in scale bar - 5 µm. Centrosomes are indicated by yellow arrow heads.

3. Choice of tissue mounting medium

To preserve stained tissue samples from drying out or deteriorating, samples were mounted in mounting medium and sealed with coverslips following immunofluorescent staining. The main factors considered for the choice of mounting medium included the refractory index of the mountant, fluorophore compatibility, specimen protection and preservation, and the absence of autofluorescent signal originating from the mounting medium. Glycerol/n-Propyl Gallate was found to allow longer-term (>1 month) storage of stained sections and visualisation of nuclei and centrosomes with minimal autofluorescence, and was therefore chosen as mounting medium for tissue sections in this study.

An example of a confocal microscopy image of a tissue stained using the above described and optimised staining workflow is shown in **Supplementary Fig. 9.**

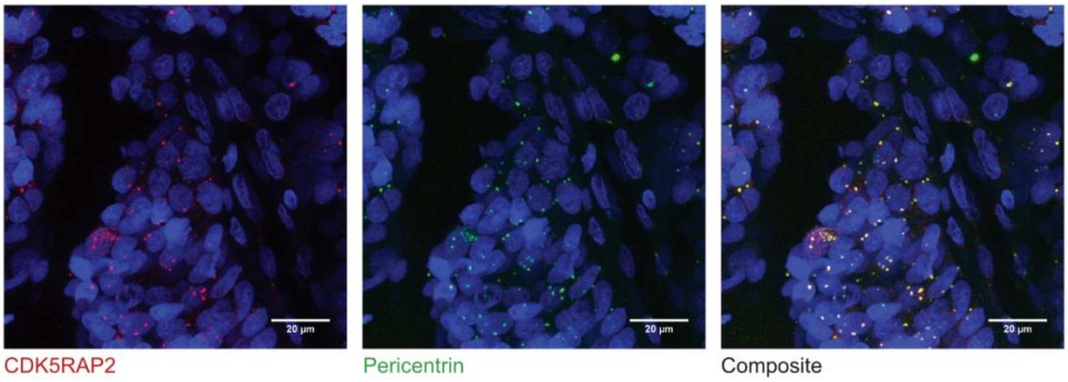

Supplementary Figure 9: Optimised FFPE tissue centrosome staining example. Confocal image (max. projection) acquired from an FFPE tissue section using the above optimised centrosome staining conditions. Hoechst is shown in blue, CDK5RAP2 is shown in red, Pericentrin is shown in green and colocalising CDK5RAP2 and Pericentrin signal is shown in yellow. Scale bar=20µm.

High-throughput microscopy image acquisition and analysis

1. Microscopy

In addition to the apparent staining challenges of centrosomes in FFPE tissue samples, three-dimensional imaging and analyses are very time-consuming and cumbersome. Imaging of centrosomes requires high imaging and optical resolution owing to their biologically small size. As a result of these limitations, previous studies investigating centrosome abnormalities in other cancer types have been limited to 3–5 imaging fields capturing only several hundreds of cells per tumour sample.

Using a manual, confocal Leica TCS SP8 microscope at 100× magnification with Z-stacks of 0.5 µm step size and three imaging channels (CDK5RAP2, Pericentrin and Hoechst), image acquisition took approximately 20–30 mins. This means that the collection of imaging data for 10 independent imaging fields would approximately take up to 5 hours for each tissue section (excluding tissue staining and image analysis time), making this approach highly challenging for larger cohort studies.

To circumvent these limitations and to enable higher throughput imaging of centrosomes in HGSOC tissues, we implemented the new Operetta CLS™ high-content analysis system. The Operetta CLS™ spinning disk technology allows high resolution imaging at fast read times and with minimal phototoxicity/photobleaching. In contrast to conventional confocal microscopy, multiple points are scanned simultaneously rather than a single point at a time, facilitating a significantly faster imaging process. An example of three adjacent tissue imaging fields acquired using the Operetta CLS™ system is shown in **Supplementary Fig. 10**.

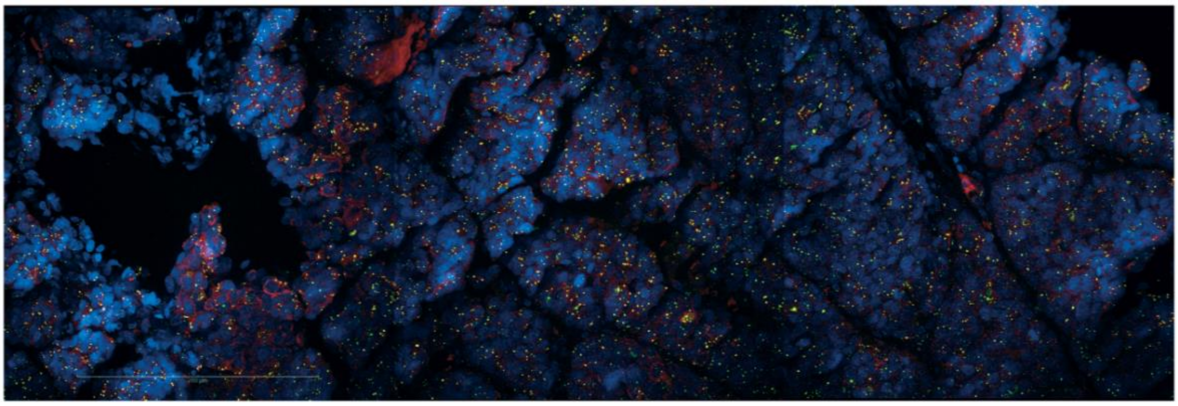

Supplementary Figure 10: Operetta CLS™ imaging example. Confocal images (max. projections) acquired from an FFPE tissue section using the above optimised centrosome staining conditions. Cells were stained for CDK5RAP2 (red), Pericentrin (green) and DNA (blue). Colocalising CDK5RAP2 and Pericentrin signal is shown in yellow. Scale bar = 200 µm.

To further automate the image acquisition process, we utilised and optimised the intrinsic Operetta PreciScan™ feature (**Supplementary Fig. 11**).

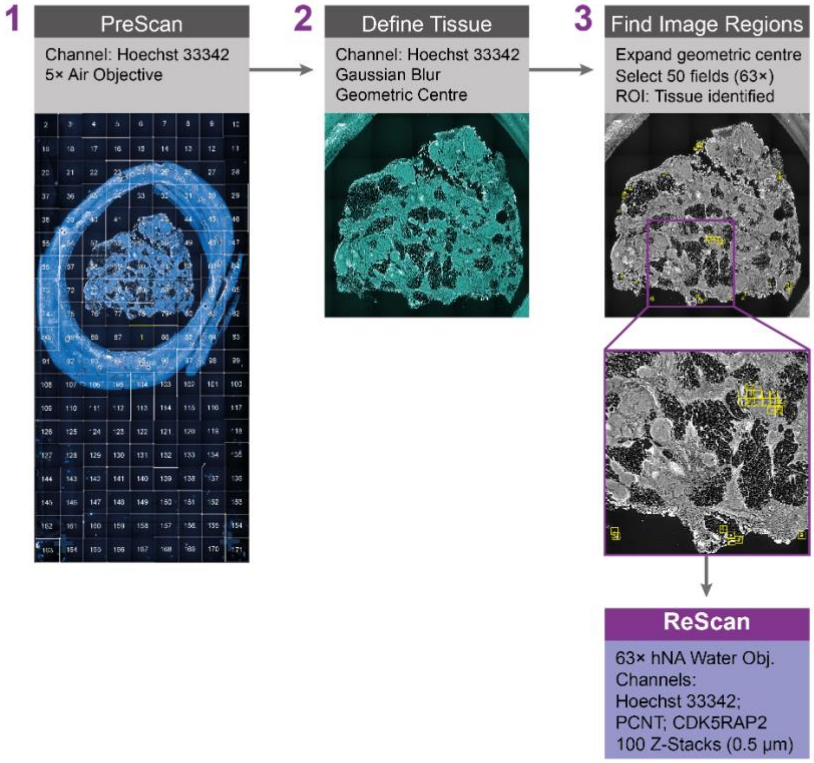

Supplementary Figure 11: PreScan workflow to identify tissue imaging fields. Flow chart representing the PreScan analysis workflow used to identify tissue imaging fields for subsequent high-resolution ReScanning.

Following low magnification (5×) imaging of whole tissue slides, global images were filtered using Gaussian blur and tissue was identified based on Hoechst 33342 signal. The geometric centre of the tissue was then calculated and expanded to create a region of interest covering the majority of the tissue. Following morphological properties calculations, 50 imaging fields were placed within the identified tissue for subsequent high resolution ReScan (63× magnification).

The PreciScan™ plug-in allows a fully automated workflow of a low magnification (5×) pre-scan, followed by an image analysis step to identify tissue regions of interest, and finally higher magnification (63×) re-scans of selected imaging fields. This approach allowed scanning of 50 independent non-overlapping imaging fields for each tissue section within a reasonable time frame, providing a larger dataset and better representation of tumour tissues.

2. Image Analysis

Images and data were reconstructed and analysed using the Harmony High-Content Analysis software. Following nuclei segmentation and spot counting, centrosome amplification (CA) scores were calculated for each of the 50 fields within a tissue sample using the following formula:

$$\text{CA Score} = \frac{\sum Centrosomes\ per\ field}{\sum Nuclei\ per\ field}$$

Supplementary Fig. 12 illustrates the workflow of the optimised image analysis sequence used for the quantification of centrosomes in HGSOC FFPE tissue samples.

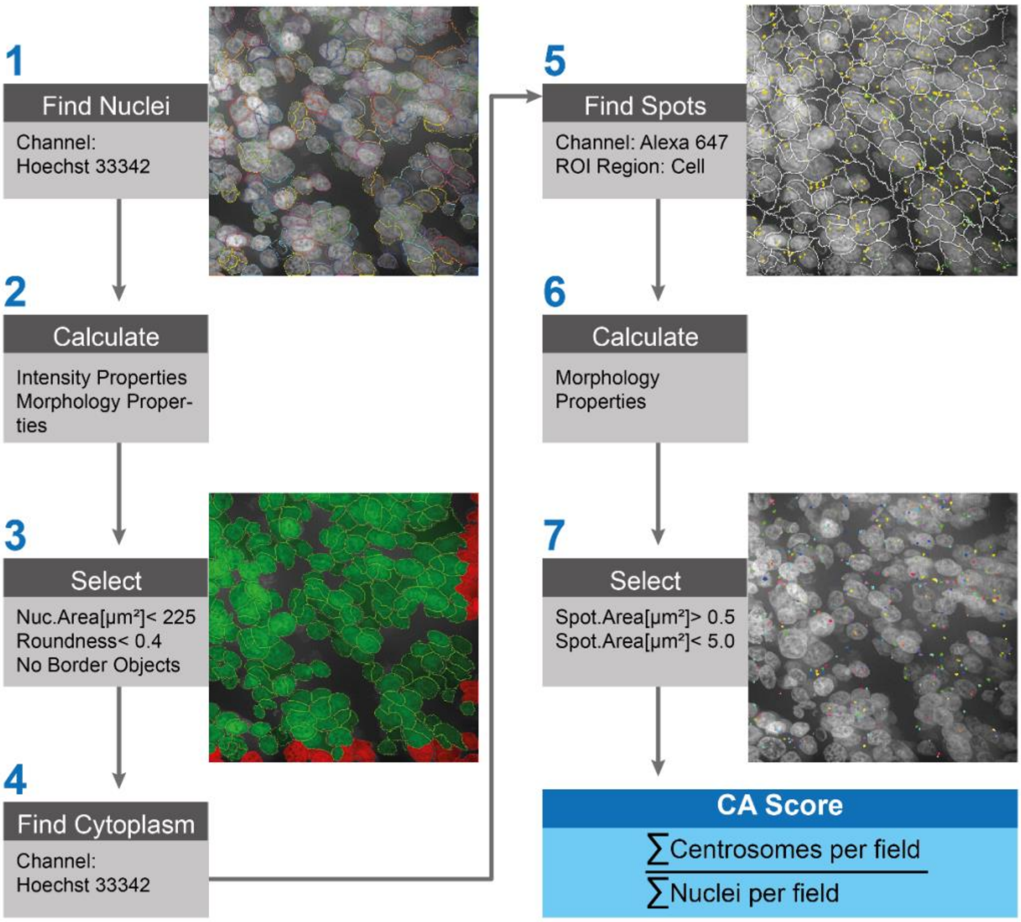

Supplementary Figure 12: Image Analysis Workflow. Flow chart representing the image analysis workflow used to identify and quantify centrosomes in human FFPE tissues. Following image acquisition, nuclei were segmented and selected based on size and roundness. Border objects were removed to only include whole nuclei during image analysis. Cytoplasm was identified based on diffusing Hoechst signal and used to identify cell boundaries. Cell

boundaries were then used as a region of interest (ROI) within which centrosomes were identified and filtered based of morphology properties. Tissue-wide CA scores were estimated as the median CA score across all imaging fields.

3. Quality control for CA analyses

The large amount of data collected (>3.5 × 10^6 images across all tissue samples) prohibited manual quality control and inspection of all imaging data. To improve the robustness of our approach, we randomly selected ten tissue sections that were stained with secondary antibodies only (no primary antibodies) to estimate background noise, autofluorescence, and the rate of false positive detection of centrosomes (**Supplementary Fig. 13a**). Based on these experiments, all imaging fields with estimated centrosome amplification scores (CA score) < 0.1 were removed from downstream analyses (**Supplementary Fig. 13b**). Inspection of these imaging fields showed that they contained images that were out of focus or captured areas of hydrophobic pen (used during antibody staining) that were misidentified as tissue during the PreScan workflow.

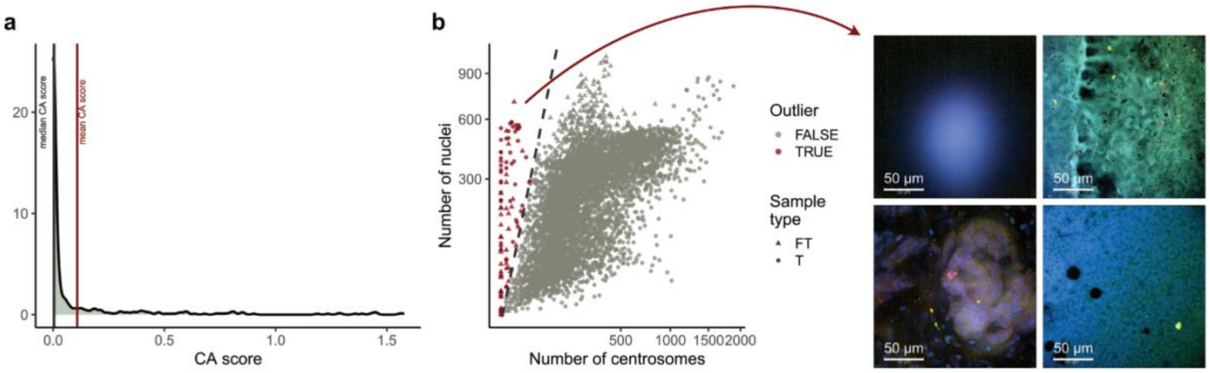

Supplementary Figure 13: Removal of outlier imaging fields. (**a**) Density plot of CA scores determined from ten control HGSOC tissue sections stained with secondary antibodies only. Median and mean CA scores across all ten sections are indicated by vertical grey and red lines, respectively. (**b**) Scatter plot showing number of nuclei vs number of centrosomes detected in each imaging fields. Low outliers are highlighted in red. Dashed diagonal line indicates CA score = 0.1. Example images from outlier imaging fields are shown on the right. T = Tumour; FT = Fallopian tube. Scale bars = 50 µm.

Supplementary References

1. De Almeida, B. P., Vieira, A. F., Paredes, J., Bettencourt-Dias, M. & Barbosa-Morais, N. L. Pan-cancer association of a centrosome amplification gene expression signature with genomic alterations and clinical outcome. *PLoS Comput Biol* **15**, 1–31 (2019).
2. Bell, D. *et al.* Integrated genomic analyses of ovarian carcinoma. *Nature* **474**, 609–615 (2011).
3. Bowtell, D. D. *et al.* Rethinking ovarian cancer II: reducing mortality from high-grade serous ovarian cancer. *Nat Rev Cancer* **15**, 668–679 (2015).
4. Duensing, S. *Analysis of centrosomes in human cancer*. *Methods in Cell Biology* vol. 129 (Elsevier, 2015).
