# Supplementary material for: Molecular landscape and functional characterization of centrosome amplification in ovarian cancer
Source: Nat Commun. 2023 Oct 16;14:6505. doi: 10.1038/s41467-023-41840-3 (PMC10579337; doi:10.1038/s41467-023-41840-3)
Supplement: Supplementary file 3 — Reporting Summary [file 41467_2023_41840_MOESM3_ESM.pdf]

nature portfolio

Corresponding author(s): Dr. Carolin M. Sauer, Prof. James D. Brenton

Last updated by author(s): Aug 22, 2023

Reporting Summary

Nature Portfolio wishes to improve the reproducibility of the work that we publish. This form provides structure for consistency and transparency in reporting. For further information on Nature Portfolio policies, see our Editorial Policies and the Editorial Policy Checklist.

Statistics

For all statistical analyses, confirm that the following items are present in the figure legend, table legend, main text, or Methods section.

n/a	Confirmed	
[ ]	[x]	The exact sample size (*n*) for each experimental group/condition, given as a discrete number and unit of measurement
[ ]	[x]	A statement on whether measurements were taken from distinct samples or whether the same sample was measured repeatedly
[ ]	[x]	The statistical test(s) used AND whether they are one- or two-sided<br>*Only common tests should be described solely by name; describe more complex techniques in the Methods section.*
[ ]	[x]	A description of all covariates tested
[ ]	[x]	A description of any assumptions or corrections, such as tests of normality and adjustment for multiple comparisons
[ ]	[x]	A full description of the statistical parameters including central tendency (e.g. means) or other basic estimates (e.g. regression coefficient) AND variation (e.g. standard deviation) or associated estimates of uncertainty (e.g. confidence intervals)
[x]	[ ]	For null hypothesis testing, the test statistic (e.g. *F*, *t*, *r*) with confidence intervals, effect sizes, degrees of freedom and *P* value noted<br>*Give P values as exact values whenever suitable.*
[ ]	[x]	For Bayesian analysis, information on the choice of priors and Markov chain Monte Carlo settings
[ ]	[x]	For hierarchical and complex designs, identification of the appropriate level for tests and full reporting of outcomes
[ ]	[x]	Estimates of effect sizes (e.g. Cohen's *d*, Pearson's *r*), indicating how they were calculated

Our web collection on statistics for biologists contains articles on many of the points above.

Software and code

Policy information about availability of computer code

Data collection

Harmony 4.9 software (PerkinElmer) was used to collect high content microscopy data.

Data analysis

High-content screening images were analysed using Harmony 4.9 software (PerkinElmer).
Sequencing data was aligned using the BWA aligner (v.0.07.17).
Additional tools/packages used: QDNAseq R package (v1.24.0); Rascal (https://github.com/crukci-bioinformatics/rascal; v0.7.0).

Downstream R packages and versions:

[1] rascal_0.7.0	shades_1.4.0	data.table_1.12.8	GGally_2.1.2	GRmetrics_1.12.2
[6] fgsea_1.12.0	Rcpp_1.0.5	DESeq2_1.26.0	SummarizedExperiment_1.16.0	DelayedArray_0.12.0
[11] BiocParallel_1.20.0	matrixStats_0.56.0	Biobase_2.46.0	GenomicRanges_1.38.0	GenomeInfoDb_1.22.0
[16] IRanges_2.20.0	S4Vectors_0.24.0	BiocGenerics_0.32.0	survminer_0.4.9	ggpubr_0.4.0
[21] survival_3.2-3	broom_0.7.0	forcats_0.5.0	stringr_1.4.0	dplyr_1.0.0
[26] purrr_0.3.3	readr_1.3.1	tidyr_1.1.0	tibble_3.0.4	ggplot2_3.3.5
[31] tidyverse_1.3.0				

Analysis code is available on GitHub: https://github.com/cmsauer/CAOV2023

For manuscripts utilizing custom algorithms or software that are central to the research but not yet described in published literature, software must be made available to editors and reviewers. We strongly encourage code deposition in a community repository (e.g. GitHub). See the Nature Portfolio guidelines for submitting code & software for further information.

Data

Policy information about availability of data

All manuscripts must include a data availability statement. This statement should provide the following information, where applicable:

- Accession codes, unique identifiers, or web links for publicly available datasets
- A description of any restrictions on data availability
- For clinical datasets or third party data, please ensure that the statement adheres to our policy

Shallow whole genome sequencing (sWGS) data and RNA sequencing data for all cell lines used in this study have been deposited to the European Nucleotide Archive (ENA) with study/project accession number PRJEB60280 (https://www.ebi.ac.uk/ena/browser/view/PRJEB60280) and is publicly accessible for download. sWGS data for OV04 tissue samples has been deposited to the European Phenome-Genome Archive (EGA) with accession number EGAD00001008121 (https://ega-archive.org/datasets/EGAD00001008121). Absolute copy number data for BriTROC-1 cases were provided by Smith et al. (2023). All genomic data relating to the BriTROC-1 study are available via EGA under accession code EGAS00001007292 (https://ega-archive.org/studies/EGAS00001007292). The genomic sequencing data for OV04 and BriTROC-1 patient samples are available under restricted access due to patient confidentiality. Access to the OV04 and BriTROC-1 sequencing data can be obtained by authorised researchers or clinicians by applying to the relevant Data Access Committees, which aim to respond to any data access applications within a week of receiving the request. There are no restrictions on the duration of access once granted. More information on EGA and on how to access data from EGA can be found here (https://www.ebi.ac.uk/training/online/courses/ega-quick-tour/accessing-the-data-in-the-ega/). Publicly available clinical data and RNAseq V2 data from The Cancer Genome Atlas (TCGA; https://www.cancer.gov/about-nci/organization/ccg/research/structural-genomics/tcga) were downloaded using Firebrowse (http://firebrowse.org/). The remaining data are available within the Article, Supplementary Information or Source Data files, and on GitHub (https://github.com/cmsauer/CAOV2023).

Human research participants

Policy information about studies involving human research participants and Sex and Gender in Research.

Reporting on sex and gender	This study utilized samples from patients with high grade serous ovarian carcinoma. Thus all participants included in this study were female.
Population characteristics	OV04: Women with cancer of the ovaries, fallopian tube or peritoneum. (Age range 44-83; median 65).<br>BriTROC-1: Women with relapsed high grade serous ovarian carcinoma that have recurred after one or more lines of platinum-containing chemotherapy. (Age range 24-85; median 65)<br>Detailed study patient and sample overview is provided in Supplementary Table 1.
Recruitment	The OV04 study is a prospective non-interventional cohort study recruiting patients with cancer of the ovary, fallopian tube or with primary peritoneal cancer. More information on recruitement is outlined here: https://www.cancerresearchuk.org/about-cancer/find-a-clinical-trial/a-study-look-more-closely-factors-affecting-how-well-chemotherapy-works-for-cancer-ovary-ov04#undefined. OV04 recruitment has started in 2008 and is currently running until March 2027.<br>The BriTROC-1 study enrolled patients with recurrent ovarian high-grade serous or grade 3 endometrioid carcinoma who had relapsed following at least one line of platinum-based chemotherapy between January 2013 and September 2017.
Ethics oversight	The OV04 study was approved by the local research ethics committee (REC reference numbers: 07/Q0106/63; and NRES Committee East of England - Cambridge Central 03/018). For BriTROC-1, Ethics/IRB approval was given by Cambridge Central Research Ethics Committee (Reference 12/EE/0349). All patients provided written informed consent.

Note that full information on the approval of the study protocol must also be provided in the manuscript.

Field-specific reporting

Please select the one below that is the best fit for your research. If you are not sure, read the appropriate sections before making your selection.

[x] Life sciences [ ] Behavioural & social sciences [ ] Ecological, evolutionary & environmental sciences

For a reference copy of the document with all sections, see nature.com/documents/nr-reporting-summary-flat.pdf

Life sciences study design

All studies must disclose on these points even when the disclosure is negative.

Sample size	No statistical method was used to predetermine clinical sample size. Instead, sample size was dictated by clinical tissue availability and suitability. Please refer to methods section "Clinical samples and primary tissue processing and selection" for further information.<br>For cell line based studies, all ovarian cancer cell lines available to us at the time were included in this study.
Data exclusions	Samples with insufficient tissue quality or purity were excluded. For association analyses between centrosome amplification and copy number data in clinical tissue samples only samples with high confidence ACN fits were included (n=54 OV04 and n=84 BriTROC samples). No data was excluded from any other analyses.

Replication	This is an observational cohort study assessing molecular landscape of supernumerary centrosomes in high grade serous ovarian carcinomas. Replications for analysis of clinical samples is not applicable in this study setting.<br>Immunofluorescent staining of cell lines and drug screens were performed in triplicates. All replications were successful.
Randomization	There was no randomisation for the treatment of ovarian cancer patients. All treatment was given at the discretion of treating oncologists. To avoid batch effects from RNA sequencing experiments, samples were randomised, taking into consideration cell line type, and growth conditions. In addition, for drug sensitivity assays, plate layouts were randomised and edge wells were left empty to prevent edge effects.
Blinding	The standard blinding is not applicable in this study considering retrospective clinical data.<br>However, blinding to the experimental data was followed during data collection and data analysis.

Reporting for specific materials, systems and methods

We require information from authors about some types of materials, experimental systems and methods used in many studies. Here, indicate whether each material, system or method listed is relevant to your study. If you are not sure if a list item applies to your research, read the appropriate section before selecting a response.

Materials & experimental systems

n/a	Involved in the study	
☐	☒	Antibodies
☐	☒	Eukaryotic cell lines
☒	☐	Palaeontology and archaeology
☒	☐	Animals and other organisms
☐	☒	Clinical data
☒	☐	Dual use research of concern

Methods

n/a	Involved in the study	
☒	☐	ChIP-seq
☒	☐	Flow cytometry
☒	☐	MRI-based neuroimaging

Antibodies

Antibodies used	All antibodies used in this study, as well as their catalog/order numbers, species, and staining concentrations, are listed in Supplementary Table 3.
Validation	Antibody validation information is available from the manufacturers (antibodies are pre-validated by manufacturer). The optimisation of antibody concentrations and microscopy assays used is described in detail in Supplementary Methods (Supplementary document, p14-19.

Eukaryotic cell lines

Policy information about cell lines and Sex and Gender in Research

Cell line source(s)	A detailed list of cell lines, RRIDs, growth conditions and providers/sources is provided in Supplementary Table 2. All cell lines used in this study were originally derived from female individuals.
Authentication	Cell line identities were confirmed prior to DNA extractions using the CRUK CI in-house human short tandem repeat (STR) profiling cell authentication service.
Mycoplasma contamination	Cells were tested for mycoplasma contaminations on a regular basis using the qPCR PhoenixDx Mycoplasma kit (Procomcure Biotech). All cell lines tested negative for mycoplasma contamination.
Commonly misidentified lines (See ICLAC register)	No commonly misidentified cell lines were used in this study.

Clinical data

Policy information about clinical studies

All manuscripts should comply with the ICMJEguidelines for publication of clinical research and a completedCONSORT checklist must be included with all submissions.

Clinical trial registration	OV04 study: A description of the clinical study is available at: https://www.cancerresearchuk.org/about-cancer/find-a-clinical-trial/a-study-look-more-closely-factors-affecting-how-well-chemotherapy-works-for-cancer-ovary-ov04#undefined<br>BriTROC-1 study: ISRCTN09180474
Study protocol	OV04 study: see above.<br>BriTROC-1 study: Protocol will be uploaded to the ISRCTN website - it is not yet uploaded but will be.
Data collection	OV04 patients enrolled between January 2010 and April 2018. See above link for further information.<br>BriTROC-1 patients were recruited from 14 UK gynaecological cancer centres between January 2013 and September 2017. More information can also be found at https://www.nature.com/articles/bjc201786#Sec2.<br>For both studies, data were collected on investigator-reported response to first study treatment following recruitment, investigator-assessed progression-free survival following first study treatment following recruitment and overall survival.
Outcomes	The OV04 study is a prospective non-interventional cohort study approved by the local research ethics committee at Addenbrooke's Hospital, Cambridge, UK, (REC reference numbers: 07/Q0106/63; and NRES Committee East of England – Cambridge Central 03/018. Further information on the OV04 study is provided in the link above.<br>The primary outcome of the BriTROC study was to obtain 300 fit-for-purpose tumour biopsies from women with relapsed HGSOC and to demonstrate the safety and feasibility of acquiring tumour biopsies from women with relapsed ovarian cancer in multiple centers. The secondary objective was to examine genomic alterations in relapsed HGSOC. More information on the BriTROC study can be found at: https://www.nature.com/articles/bjc201786#Sec2 and https://www.nature.com/articles/bjc201786#Sec2.
